# Destabilization of the D2 domain of *Thermotoga maritima* arginine binding protein induced by guanidinium thiocyanate and its counteraction by stabilizing agents

**DOI:** 10.1002/pro.5146

**Published:** 2024-08-16

**Authors:** Guido Izzi, Antonella Paladino, Rosario Oliva, Giovanni Barra, Alessia Ruggiero, Pompea del Vecchio, Luigi Vitagliano, Giuseppe Graziano

**Affiliations:** ^1^ Department of Chemical Sciences University of Naples Federico II Naples Italy; ^2^ Institute of Biostructures and Bioimaging, CNR Naples Italy; ^3^ Department of Science and Technology University of Sannio, via Francesco de Sanctis snc Benevento Italy

**Keywords:** calorimetry, Guanidinium, MD simulations, thermodynamics, thiocyanate, TmArgBP

## Abstract

D2 is a structural and cooperative domain of *Thermotoga maritima* Arginine Binding Protein, that possesses a remarkable conformational stability, with a denaturation temperature of 102.6°C, at pH 7.4. The addition of potassium thiocyanate causes a significant decrease in the D2 denaturation temperature. The interactions of thiocyanate ions with D2 have been studied by means of isothermal titration calorimetry measurements and molecular dynamics simulations. It emerged that: (a) 20–30 thiocyanate ions interact with the D2 surface and are present in its first solvation shell; (b) each of them makes several contacts with protein groups, both polar and nonpolar ones. The addition of guanidinium thiocyanate causes a marked destabilization of the D2 native state, because both the ions are denaturing agents. However, on adding to the solution containing D2 and guanidinium thiocyanate a stabilizing agent, such as TMAO, sucrose or sodium sulfate, a significant increase in denaturation temperature occurs. The present results confirm that counteraction is a general phenomenon for globular proteins.

## 1 INTRODUCTION

The Arginine Binding Protein from *Thermotoga maritima*, TmArgBP, is a special protein as it is endowed with an extraordinary stability to pressure (Jaworek et al., 2020), temperature (Luchansky et al., 2009) and chemical denaturants (Ruggiero et al., 2014). Moreover, it has a dimeric structure (each monomer consists of 246 residues), as a consequence of the domain swapping of the C‐terminal helix between two monomers, as demonstrated by the X‐ray structure (Ruggiero et al., 2014). TmArgBP is very resistant to temperature, with a denaturation temperature around 115°C (Smaldone et al., 2016), well above the normal boiling temperature of water, notwithstanding the not‐so‐compact organization of the swapped dimeric structure. In order to shed light on its peculiar features, TmArgBP has been dissected in different ways. By eliminating the swapping C‐terminal helix (residues 232–246), a monomeric form has been obtained and its detailed characterization has been performed (Smaldone et al., 2018). In addition, two domains, corresponding to the two halves of the monomer, have been engineered, termed D1 and D2, respectively (Smaldone et al., 2018). The latter consists of 92 residues, its crystal structure shows the presence of a large fraction of both α‐helices and β‐sheet secondary structure elements. D2 is not able to bind arginine, but has remarkable stability against temperature; DSC measurements indicated that its temperature‐induced denaturation is a two‐state reversible process with a denaturation temperature of about 102°C (Smaldone et al., 2018). These features render the D2 domain a suitable model to perform investigations on the subtleties of the conformational stability of globular proteins, especially those of hyper‐thermophilic origin.

It is well established that some ions have a strong denaturing effect toward the native state of globular proteins (Baldwin, 1996; Collins & Washabaugh, 1985); they are classified as “salting‐in” agents, according to the Hofmeister series (Hofmeister, 1888). Thiocyanate is one of the strongest “salting‐in” anions, and guanidinium is one of the strongest “salting‐in” cations. A molecular level rationalization of the Hofmeister series is still lacking, and represents the target of several research investigations (Gregory et al., 2022; Zhang & Cremer, 2010). Some of us have recently shown that the protein structures deposited in the PDB offer the opportunity to “see” the direct interactions of the most common denaturing agents, urea, guanidinium, and thiocyanate ions, with the protein surfaces (Cozzolino et al., 2020; Paladino, Balasco, Graziano, & Vitagliano, 2022; Paladino, Balasco, Vitagliano, & Graziano, 2022). A complete survey of the PDB structures has revealed that these denaturants make direct interactions with proteins since it has been possible to structurally characterize true binding sites. It emerged that both guanidinium and thiocyanate ions make a lot of contacts, six on average, with both polar and nonpolar moieties (i.e., they are very promiscuous) (Paladino, Balasco, Graziano, & Vitagliano, 2022). These findings pushed us to investigate, both experimentally and computationally, the effect that thiocyanate alone or thiocyanate and guanidinium together (i.e., the guanidinium thiocyanate salt, GdmSCN) can have on a very stable small globular protein such as the D2 domain of TmArgBP. Finally, the simultaneous action of denaturing and stabilizing agents on D2 has been investigated.

## 2 RESULTS AND DISCUSSION

### 2.1 Thiocyanate binding to D2: DSC measurements

**FIGURE 1:**
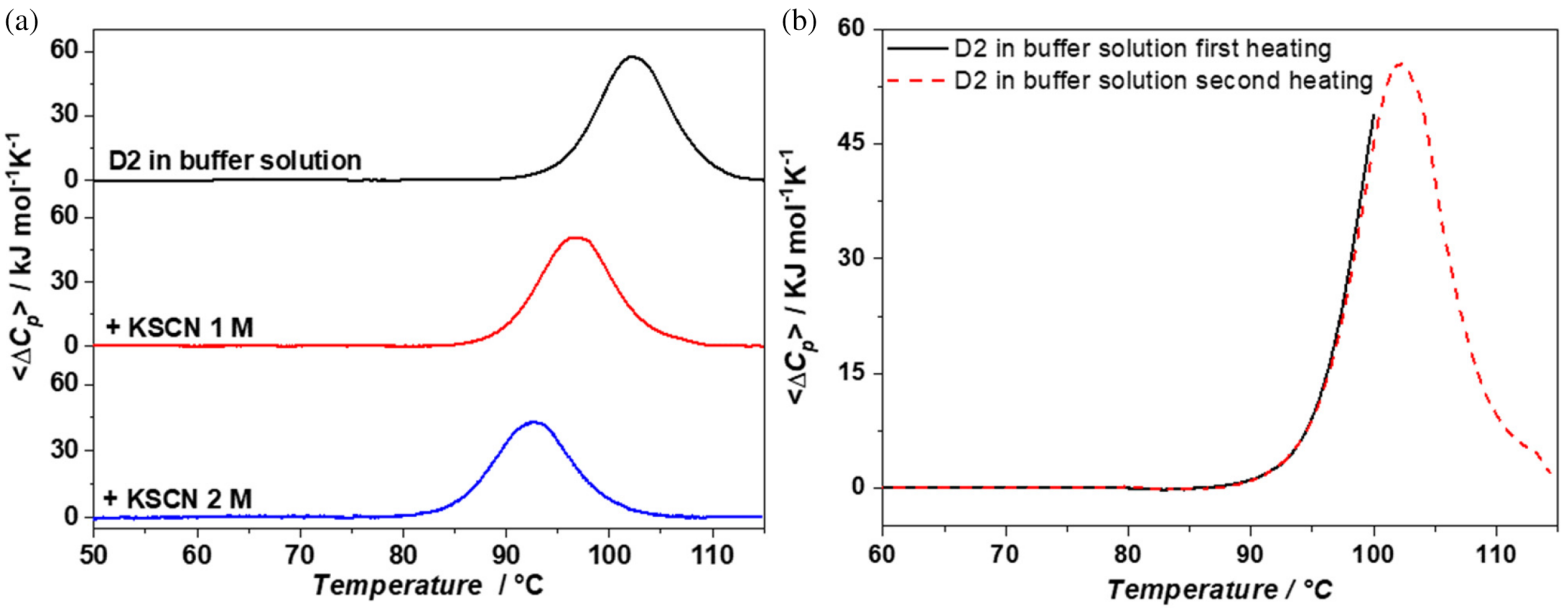
DSC traces of D2 in aqueous 20 mM phosphate buffer, pH 7.4, and in the presence of different KSCN concentrations (a); DSC profiles of the first heating (black solid line) and second heating (red dashed line) of D2 in aqueous buffer solution (b).

DSC measurements indicate that, at pH 7.4, 20 mM phosphate buffer, the D2 domain has a denaturation temperature above the normal boiling temperature of water, *T*_*d*_ = 102.6°C. This remarkable thermal stability is confirmed by DSC measurements performed in the presence of increasing concentrations of KSCN (see Figure 1a, and part A of Table 1). At 1 M KSCN, *T*_*d*_ = 97.0°C, and at 2 M KSCN, *T*_*d*_ = 92.6°C. The temperature‐induced denaturation proves to be reversible. The re‐heating criterion has been used to assess the reversibility with a special care: it is necessary to stop the first heating at 100°C, to cool the sample to 25°C, and then to re‐heat up to 115°C. Using this procedure, the temperature‐induced denaturation of D2 proves to be reversible; see Figure 1b. This care is necessary because the time spent by the protein above 100°C is a critical factor for the chemical stability of the chain. The process is well described by the reversible two‐state transition model, since the value of the calorimetric to van't Hoff enthalpy ratio is close to one (see the numbers listed in the last column of Table 1). In a previous study, it was shown that, at pH 7.4, 20 mM phosphate buffer, and increasing concentrations of GdmCl, the temperature‐induced denaturation of D2 is well described by the reversible two‐state transition model, with *T*_*d*_ = 94.0°C at 1 M GdmCl, and *T*_*d*_ = 86.2°C at 2 M GdmCl (Cozzolino et al., 2020); see part C in Table 1. DSC data indicate that the guanidinium ion is a stronger destabilizing agent of the D2 native state in comparison to the thiocyanate ion.

**TABLE 1:** Thermodynamic parameters from the analysis of DSC curves for the temperature‐induced denaturation of D2 domain in the absence and presence of different stabilizing and destabilizing agents, at pH 7.4, 20 mM phosphate buffer.

Cosolute		*T*_*d*_ (°C)	∆*H*_*d*_(*T*_*d*_) (kJ mol^−1^)	∆*H*_*d*_(*T*_*d*_)^vH^ (kJ mol^−1^)	∆*H*_*d*_(*T*_*d*_)/∆*H*_*d*_(*T*_*d*_)^vH^
–	–	102.6 ± 0.2	470 ± 24	490	0.96
(A) KSCN					
0.5 M	–	99.6 ± 0.2	470 ± 24	480	0.98
1.0 M	–	97.0 ± 0.2	436 ± 22	450	0.97
2.0 M	–	92.6 ± 0.2	425 ± 21	435	0.98
(B) GdmSCN					
0.5 M	–	90.2 ± 0.2	405 ± 20	409	0.99
1.0 M	–	78.4 ± 0.2	341 ± 17	354	0.96
1.5 M	–	64.5 ± 0.2	256 ± 13	265	0.97
2.0 M	–	50.4 ± 0.2	212 ± 11	223	0.95
(C) GdmCl					
0.5 M		98.2 ± 0.3	440 ± 24	458	0.96
1.0 M		94.0 ± 0.3	430 ± 21	439	0.98
2.0 M		86.2 ± 0.3	370 ± 19	390	0.95
(D) GdmSCN					
1 M	1 M TMAO	84.5 ± 0.2	417 ± 21	426	0.98
1 M	1 M sucrose	85.0 ± 0.2	405 ± 20	412	0.98
1 M	0.5 M TMAO + 0.5 M sucrose	84.6 ± 0.2	417 ± 21	424	0.98
1 M	0.5 M Na_2_SO_4_	91.1 ± 0.2	420 ± 21	415	1.01
1 M	1 M Na_2_SO_4_	99.6 ± 0.2	417 ± 21	427	0.97

### 2.2 Thiocyanate binding to D2: ITC measurements

A survey of protein structures deposited in the PDB has revealed the existence of a large number of crystal structures containing the SCN^−^ ion (Paladino, Balasco, Graziano, & Vitagliano, 2022). Careful analysis of such structures has allowed the characterization of 712 different binding sites of SCN^−^ ion on protein surfaces. It emerged that SCN^−^ is very promiscuous, being able to make contacts with all types of protein moieties, both main chain and side chains (Paladino, Balasco, Graziano, & Vitagliano, 2022). This important and somewhat unexpected feature should be a key factor for its denaturing power (actually, a feature shared with guanidinium ions and also urea molecules) (Paladino, Balasco, Vitagliano, & Graziano, 2022).

**FIGURE 2:**
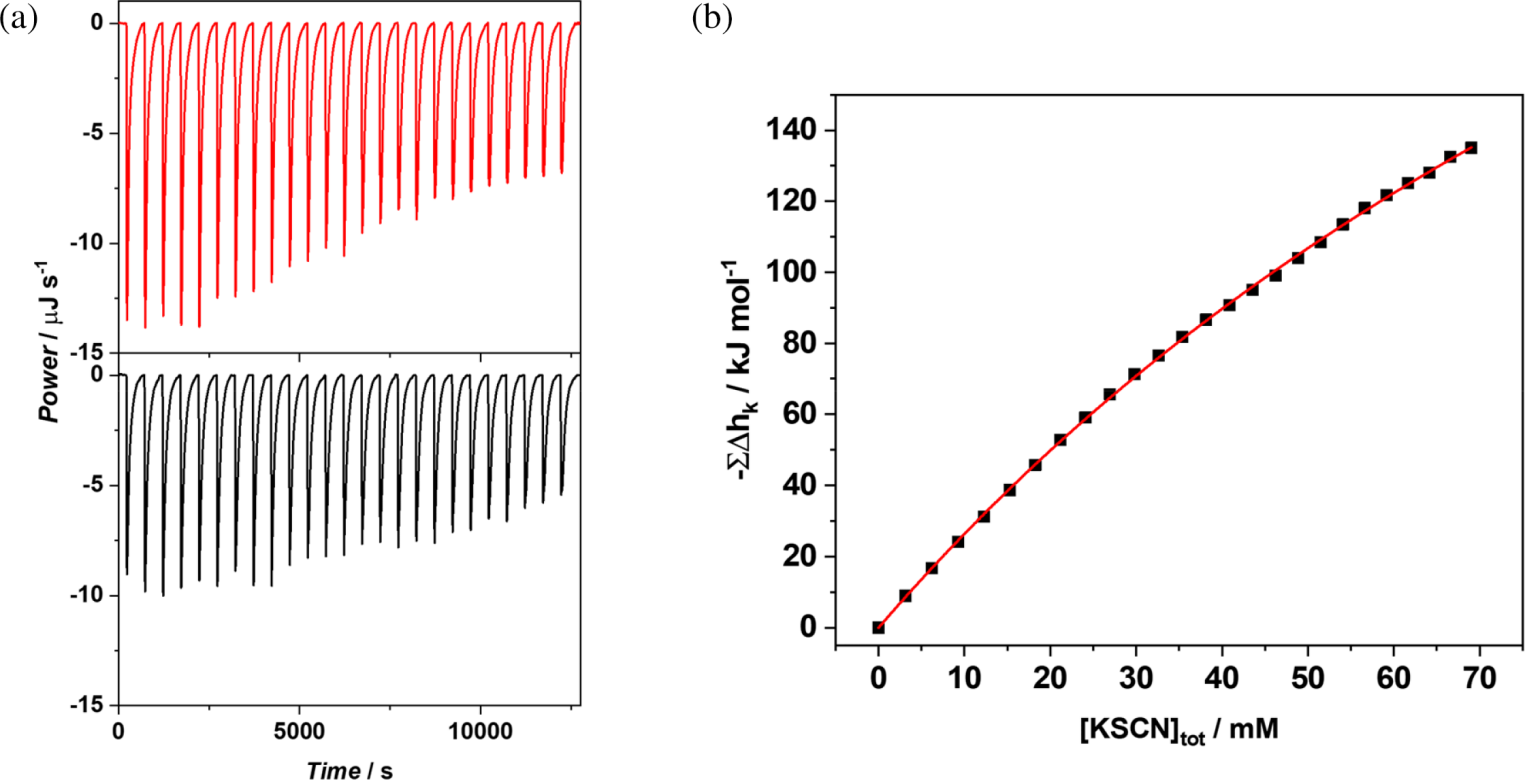
ITC trace obtained from the titration of a D2 solution (42 μM) with a solution of KSCN 300 mM; the black line is the raw ITC trace obtained from the binding experiment; the red line is the raw ITC trace obtained from the KSCN dilution experiment (a); binding isotherm for the interaction between SCN^−^ and D2: The black squares represent the cumulative heats of interaction obtained from the ITC traces reported in panel a; the red line is the best fit of experimental data according to an independent and equivalent binding site model with a stoichiometry *n* = 30 ± 10. The experiment was performed in duplicate at the temperature of 25°C in 20 mM phosphate buffer, pH 7.4 (b).

**FIGURE 3:**
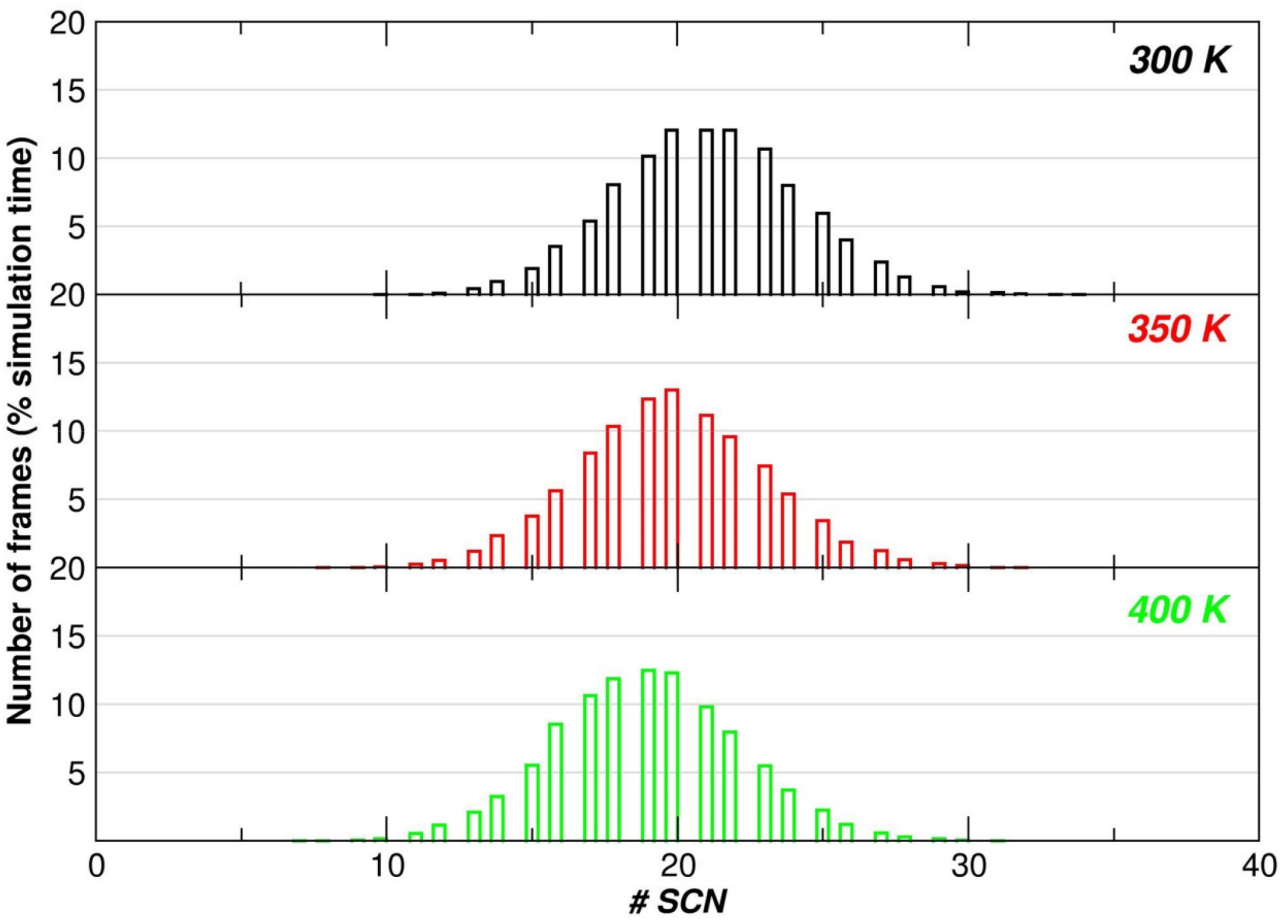
Percentage fraction of frames in which a given number of SCN^−^ ions occurs within a shell of 4 Å thickness around the protein surface at 300, 350, and 400 K, respectively, during the entire simulation time (i.e., 1 μs by considering all the 5 MD trajectories performed at each temperature, 10,000 analyzed frames). The obtained mean values are 21.1, 19.9, and 19.0 passing from 300 to 350 K and 400 K, respectively.

In order to verify the ability of SCN^−^ to favorably interact with the D2 domain, ITC measurements have been performed at 25°C, by titrating the protein with an aqueous solution of KSCN. ITC data indicate that the interaction between SCN^−^ and D2 is characterized by a low binding enthalpy change because the calorimetric peaks recorded from the titration are comparable to those obtained from the SCN^−^ dilution (see Figure 2a). After subtraction of the heat of KSCN dilution, the obtained data were analyzed by best fitting the cumulative measured heat versus the KSCN concentration (whose value in the sample cell increases during the titration), in the assumption that SCN^−^ association occurs to a fixed number of equal and independent binding sites. The fit is satisfactory (see Figure 3), and the results are: the number of bound SCN^−^ ions is *n* = 30 ± 10, the binding constant per site is *K*_*b*_ = 6.3 ± 0.2 M^−1^, and the overall binding enthalpy change is Δ*H*_*b*_ = −14.9 ± 5.0 kJ mol^−1^. These values indicate that several SCN^−^ ions bind to practically equal and independent sites, with a very small binding constant and a small negative enthalpy change per site.

Note that similar values for the binding constant of the SCN^−^ ions to elastin‐like polypeptides and to poly(N‐isopropylacrylamide) (PNIPAM) have been obtained by means of an analysis of its effect on the collapse temperature of the polymers. Specifically, at room temperature, Cremer and colleagues obtained that *K*_*b*_ is in the range 3.7–6.7 M^−1^ for the SCN^−^ binding to elastin‐like polypeptides, and *K*_*b*_ = 4.3 M^−1^ for the binding to PNIPAM (Rembert et al., 2012; Zhang et al., 2005). Moreover, electrophoretic NMR measurements, at room temperature, led to *K*_*b*_ = 6.7 M^−1^ for the binding to PNIPAM (Fang & Furó, 2021). The scenario is in line with that emerged from the analysis of PDB structures and the statistical thermodynamic models devised to rationalize experimental data (Paladino, Balasco, Graziano, & Vitagliano, 2022; Paladino, Balasco, Vitagliano, & Graziano, 2022). Of course, the denaturation mechanism cannot be understood by looking at a single binding site, it is necessary to consider all the binding sites, also the ones “created” by chain unfolding. Direct interaction of SCN^−^ ions to protein surface (with concomitant displacement of some water molecules) is a necessary condition to enhance association on increasing the SCN^−^ concentration, and to produce a sizeable Gibbs free energy decrease (due to the negative enthalpy change and the entropy gain associated with the large number of different configurations produced by the occupied and unoccupied sites on protein surface, with the caveat that both quantities depend on temperature) (Paladino, Balasco, Vitagliano, & Graziano, 2022).

### 2.3 Thiocyanate binding to D2: MD simulations

**FIGURE 4:**
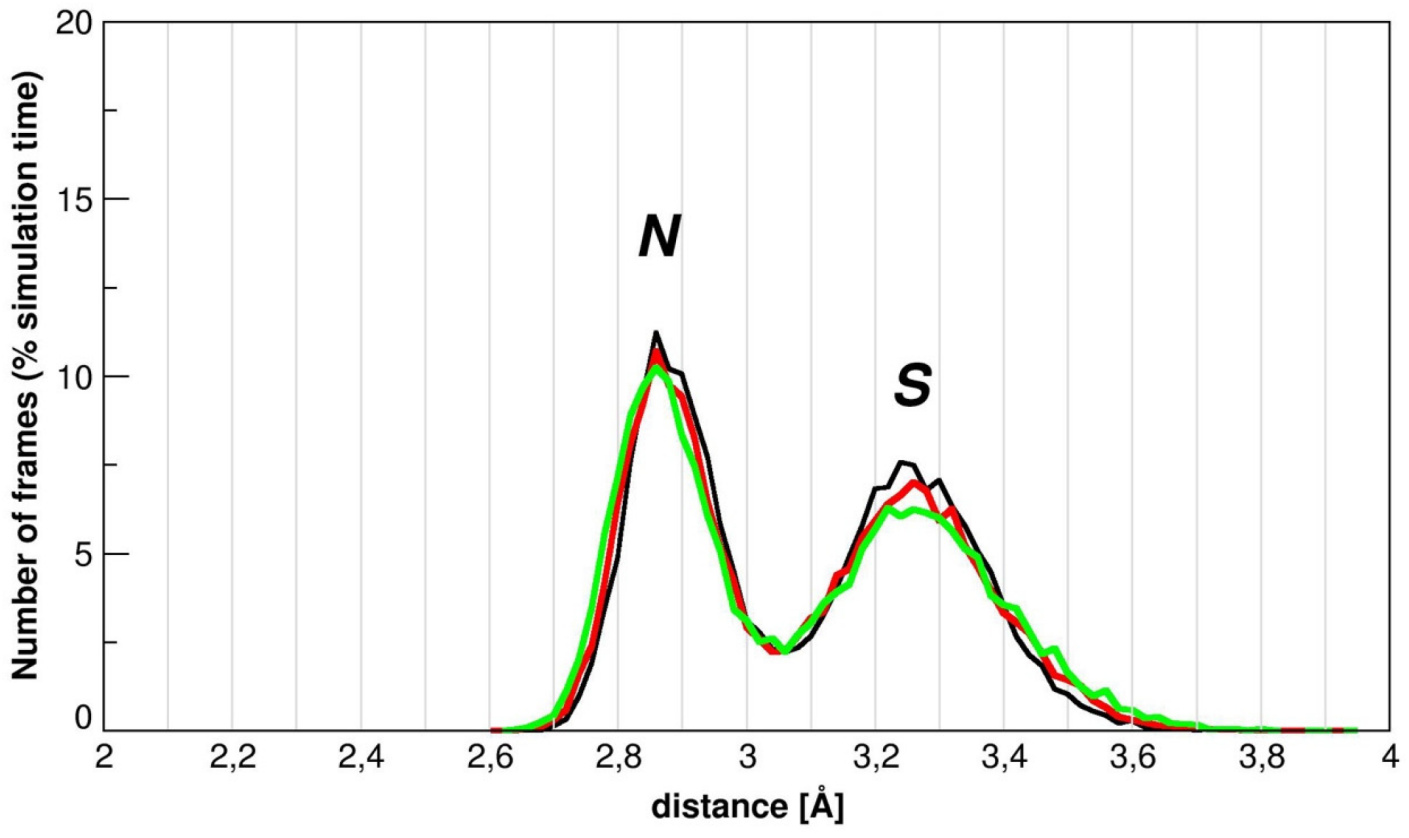
Minimal distance distribution between thiocyanate atoms and D2 surface atoms. S and N distances are reported at 300, 350, and 400 K. Protein hydrogens are not considered in the calculation.

In order to gain structural information on the thiocyanate binding to D2, crystallization trials were performed in the presence of the denaturant. Although the crystals diffract at high resolution (~1.5 Å), the diffraction pattern is indicative of the presence of multiple/disordered crystals, a feature that was also evident from the inspection of the crystal morphology (see Figure S1). Based on these results, to directly “see” the presence of SCN^−^ ions on the D2 surface, MD trajectories, each 200 ns long, have been run at 300, 350, and 400 K, for two different systems. In the first case, a single D2 molecule was immersed in a box containing water; in the second case, a single D2 molecule was immersed in a 2 M KSCN aqueous solution. Five MD trajectories have been run for each system and each temperature; so 1 μs of simulations has been done at each temperature, for each system. The D2 domain proves to be conformationally stable in all the trajectories, with Rg values always around 10.8 Å, in both water and 2 M KSCN, at 300 K (see Figure S2), and moderate side chain fluctuations (with residues *rms* deviations <1 Å, Figure S2C). Analysis of the 5 MD trajectories indicates that on average 20 SCN^−^ ions are within a shell of 4 Å thickness around the protein surface during half the simulation time (>0.5 μs) and at all three temperatures (see the histograms in Figure 3). In addition, the number of such SCN^−^ ions is no <10 *per* simulation time frame, in line with ITC results and expectations grounded in the results of the PDB survey (Paladino, Balasco, Graziano, & Vitagliano, 2022). Thiocyanate ions can establish several types of interactions: although most of the contacts are made with polar side chains, preferably positively charged residues of the protein surface, SCN^−^ mediates a number of interactions with nonpolar moieties (see Table 2). Moreover, the analysis of the minimum distance distribution between thiocyanate and D2 surface atoms reveals a peak around 2.9 Å in the case of nitrogen, and a peak around 3.25 Å in the case of sulfur (see Figure 4). These distance values indicate that both nitrogen and sulfur atoms of thiocyanate make H‐bonds with protein groups. A similar scenario emerged for the interactions of the guanidinium ions with the D2 surface (Cozzolino et al., 2020).

**TABLE 2:** Statistics of the contacts between SCN^−^ ions and D2 along the entire simulation time (i.e., 1 μs considering all the 5 MD trajectories at each temperature)

	Positive *side chains* 8 Lys, 6 Arg		Negative *side chains* 10 Asp, 3 Glu		Aromatic *side chains* 5 Phe, 2 Tyr		Aliphatic *side chains 14 Val, 5 lle, 6 Leu, 8 Ala*	
	min; max	<contacts>	min; max	<contacts>	min; max	<contacts>	min; max	<contacts>
300 K	23; 214	104	0; 47	7	0; 57	17	3; 93	38
350 K	17; 221	94	0; 32	6	0; 57	15	0; 87	32
400 K	10; 206	91	0; 32	5	0; 51	11	0; 86	26

*Note*: Minimum and maximum contact number (min; max) and average number of contacts (<contacts>) are reported *per* amino acid group. The number of amino acid type in each group is indicated. Only side chains mediated contacts are considered; glycine and proline residues are not considered.

**FIGURE 5:**
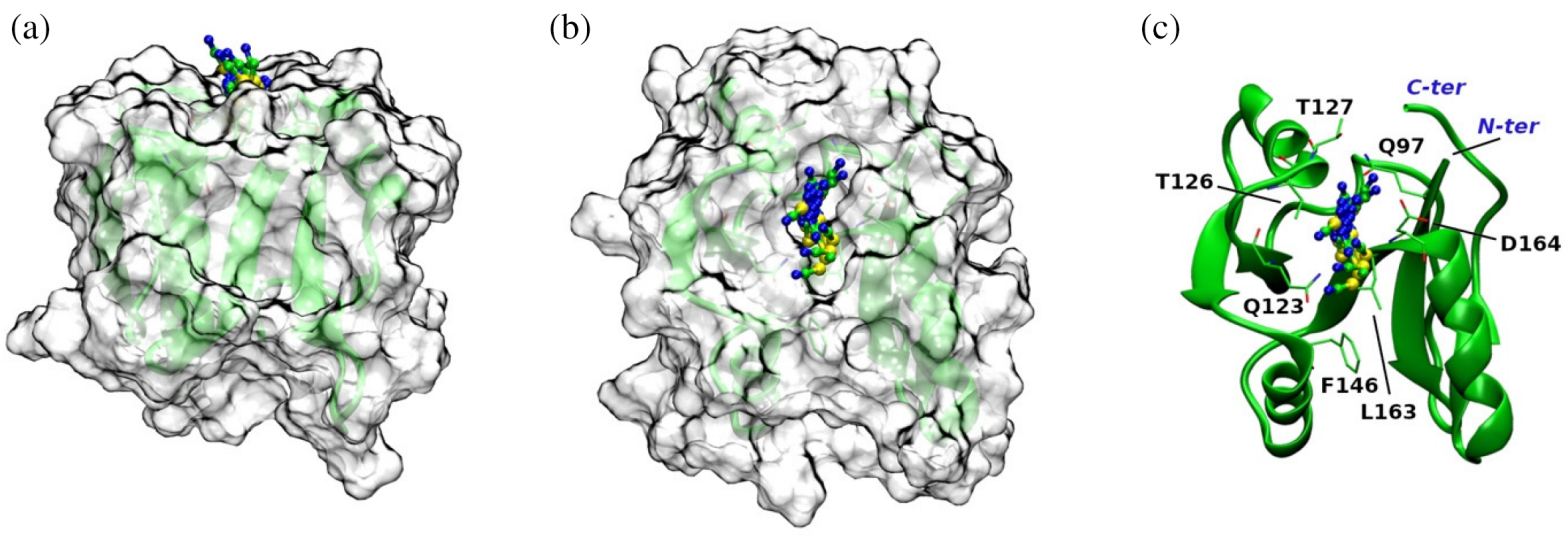
Example of a persistent SCN^−^—binding site in D2: Ghost surface representation is used to localize the binding pocket onto the protein, while ball‐and‐sticks are used for the SCN^−^ ion; SCN^−^ poses, corresponding to different time frames from a single MD run, are reported (a and b); close‐up view of the binding site: Amino acid side chains that stabilize the SCN^−^ ion are labeled and displayed in sticks; spheres are used for SCN^−^ (S = yellow, C = green, N = blue) and the protein is rendered in green cartoons (c).

**FIGURE 6:**
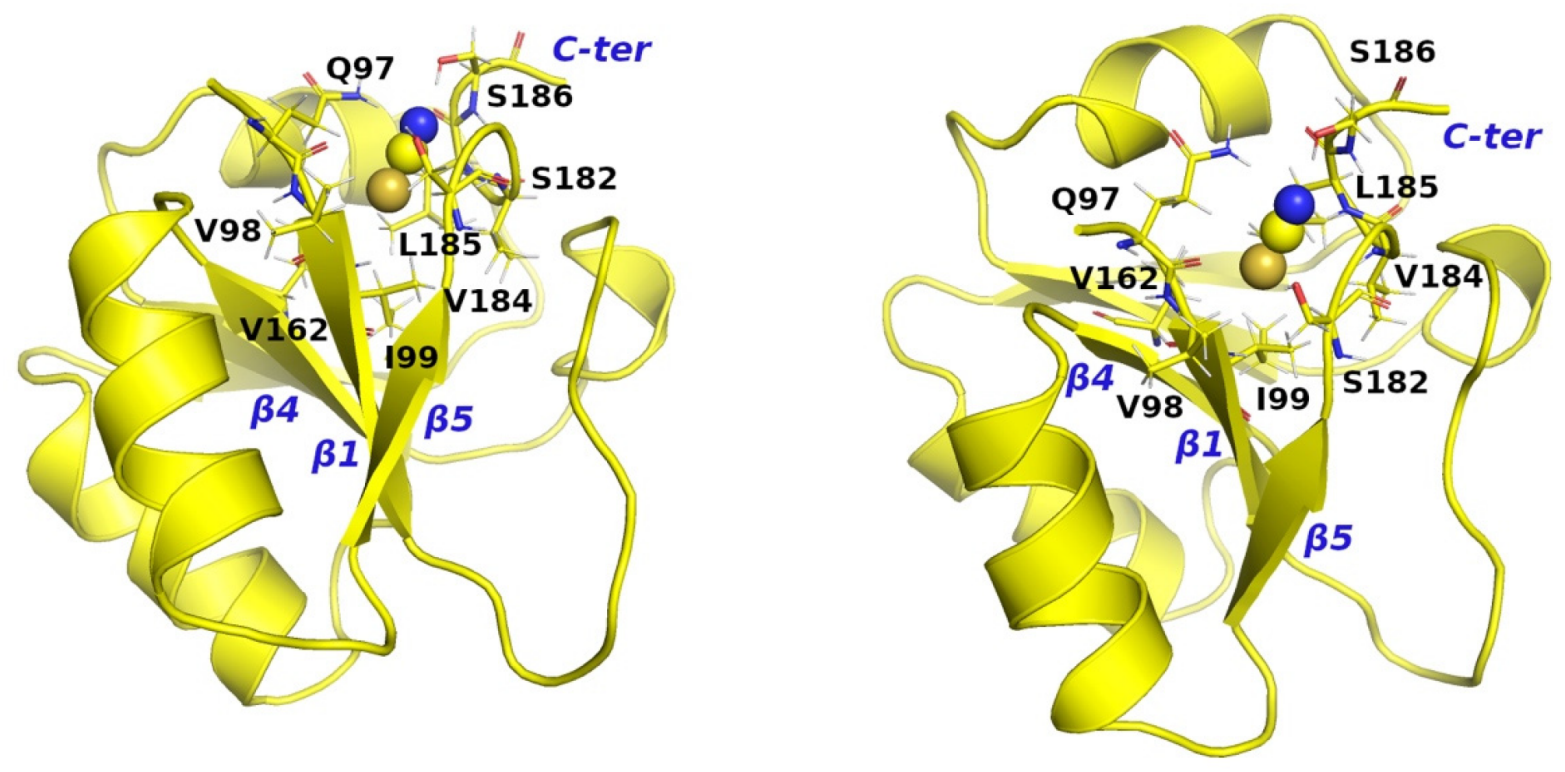
Emergence of a “new” SCN^−^ binding site during the MD trajectories at 400 K; two different orientations are provided. Amino acid side chains that stabilize the SCN^−^ ion are labeled and displayed in sticks; spheres are used for SCN^−^ (S = brown, C = yellow, N = blue); the protein is rendered in yellow cartoons.

MD data show that interactions with all kinds of protein moieties decrease at higher temperatures, yet the number of contacts established with surface arginines and lysines decreases to a lesser extent. The impact of the temperature relies on protein groups and the contacts established by SCN^−^ ions with positive‐charged residues show a ~1.1 drop ratio compared to a ~1.5 drop for the other amino acid groups (see Table 2). Except for minimal adjustments, high‐temperature simulations confirmed the binding propensities of SCN^−^ ions to the protein surface. The observed binding events appear to be fast and exchangeable, allowing, on average, about 20 ions to simultaneously bind the D2 surface, establishing both electrostatic and nonpolar interactions (see Figure 5). On increasing the temperature, these interactions undergo large fluctuations, and only a few common, and somehow *persistent*, binding pockets can be identified (see Figure 6). By comparing the D2 surface patches interacting with SCN^−^ ions at different temperatures, it is possible to detect some loci that become suitable for SCN^−^ interaction only at 400 K (i.e., upon initial unfolding). Interestingly, even though observed only for a marginal timescale (5% of a 200 ns MD run), these side chains make a novel SCN^−^ binding pocket, otherwise out of reach. Figure 6 shows the novel SCN^−^ binding site enclosed by β1/β4/β5 strands of the β‐sheet and the C‐terminal region of the protein. It is worth underlining that most of the groups interacting with the ion are nonpolar side chains, quite buried in the folded protein core.

### 2.4 Action of thiocyanate and guanidinium ions on D2

**FIGURE 7:**
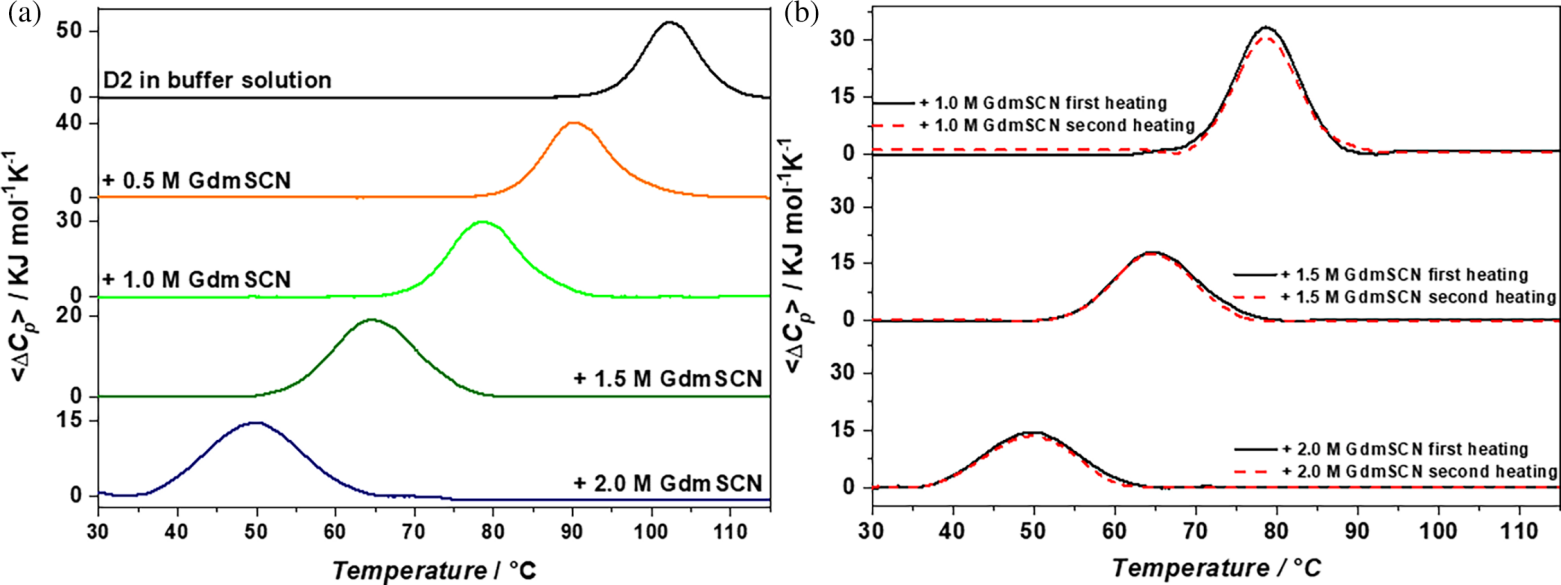
DSC traces of D2 in aqueous 20 mM phosphate buffer, pH 7.4, in the presence of different GdmSCN concentrations (a); DSC traces of the first heating (black solid line) and second heating (red dashed line) for D2 in buffer solution containing 1, 1.5 and 2 M GdmSCN (b).

The combined action of thiocyanate and guanidinium was investigated by treating the D2 domain with GdmSCN. DSC measurements at pH 7.4, 20 mM phosphate buffer, in the presence of increasing GdmSCN concentrations, demonstrate that the combined action of the two destabilizing agents has a marked effect on the protein conformational stability. Indeed, even though the temperature‐induced denaturation is reversible according to the re‐heating criterion, and well represented by the reversible two‐state transition model (see the values of the calorimetric to van't Hoff enthalpy ratio in the last column of Table 1), *T*_*d*_ = 78.4°C at 1 M GdmSCN, and *T*_*d*_ = 50.4°C at 2 M GdmSCN; see Figure 7a,b, and part B in Table 1. The strong drop in the T_d_ value of the D2 domain by more than 50 Celsius degrees, on passing from the aqueous buffer to the 2 M GdmSCN aqueous solution, has to be put in the right perspective. It is necessary to recognize that GdmSCN is a very strong denaturing agent (i.e., both the guanidinium and thiocyanate ions have a destabilizing action), and that common globular proteins are unfolded in 2 M GdmSCN aqueous solution, at room temperature (Sengupta et al., 2016; Stepanenko et al., 2012; Von Hippel & Wong, 1965).

In general, the denaturant addition causes a decrease in the denaturation enthalpy change of D2: Δ*H*_*d*_(*T*_*d*_) = 470 kJ mol^−1^ in aqueous buffer, 425 kJ mol^−1^ in 2 M KSCN, 370 kJ mol^−1^ in 2 M GdmCl, and 212 kJ mol^−1^ in 2 M GdmSCN (see the numbers listed in the fourth column of Table 1). It is evident that the combined action of the guanidinium and thiocyanate ions has a marked effect on the Δ*H*_*d*_(*T*_*d*_) magnitude. A least‐squares linear regression of the Δ*H*_*d*_(*T*_*d*_) values versus the *T*_*d*_ values of all the DSC measurements of D2 in aqueous solution and in the presence of GdmCl, KSCN, and GdmSCN (i.e., 13 points) produces a slope equal to 5.2 ± 0.3 kJ K^−1^ mol^−1^, with *R* ^2^ = 0.9658 (plot not shown). The slope is an estimate of Δ*C*_*p,d*_, the change in heat capacity upon denaturation, even though it should be recognized that the presence of denaturants could have a significant effect (note that the Δ*C*_*p,d*_ values obtained directly from DSC scans show large differences among each other, and it is meaningless to make an average of them). The obtained value is close to estimates calculated by means of empirical relationships based on the assumption of a linear dependence of Δ*C*_*p,d*_ on the number of residues constituting the globular protein (Robertson & Murphy, 1997; Sawle & Ghosh, 2011). Using the above Δ*C*_*p,d*_ value, considered to be temperature‐independent, it is simple to calculate Δ*H*_*d*_ at 60°C and to make a comparison with the average value obtained for mesophilic globular proteins, 2.92 kJ mol^−1^ per residue (Auton et al., 2011). The exercise leads to 2.70 kJ mol^−1^ per residue in the case of D2, a quantity smaller than the average value for mesophilic globular proteins. This finding is in line with the emerging evidence that the extra‐thermal stability of thermophilic globular proteins is not due to enthalpic (energetic) factors, but to entropic ones (Karshikoff et al., 2015; Khechinashvili et al., 2008; Pica & Graziano, 2016; Sawle & Ghosh, 2011).

**FIGURE 8:**
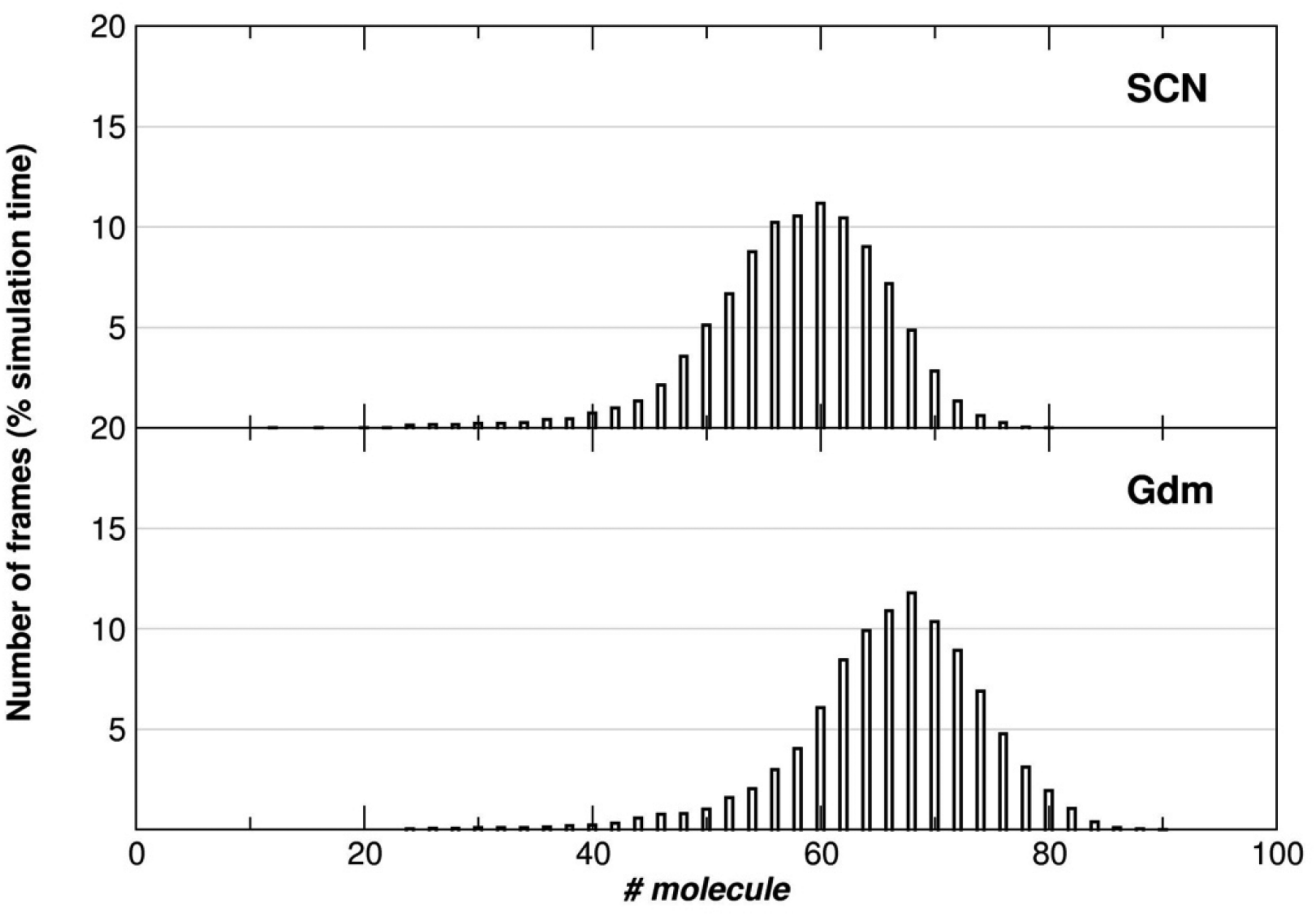
Percentage fraction of frames in which a given number of SCN^−^ ions or of Gdm^+^ ions occurs within a shell of 4 Å thickness around the protein surface at 300 K, during the entire simulation time (i.e., 1 μs by concatenating the 5 MD trajectories, 10,000 analyzed frames). The obtained mean values are 57.4 for SCN^−^ ions and 65.6 Gdm^+^ ions.

The combined effect of the guanidinium and thiocyanate ions has been addressed by MD studies carried out on the D2 domain in 2 M GdmSCN aqueous solution. MD outcomes highlight a larger number of both Gdm^+^ and SCN^−^ ions within a shell of 4 Å thickness around the protein, compared to the results obtained from MD trajectories in 2 M KSCN. Analysis of ion distribution around the protein surface, indicates that for a very long‐lasting window (i.e., >500 ns) ~66 Gdm^+^ ions and ~57 SCN^−^ ions are within a shell of 4 Å thickness around the protein surface (look at Figure 8). It is worth underscoring that these values correspond approximately to a three‐fold increase compared to those found in MD trajectories carried out in 2 M KSCN, and provide a rough explanation of the measured synergic effect when both guanidinium and thiocyanate ions are present in the aqueous solution.

### 2.5 General counteraction of stabilizing agents on D2

**FIGURE 9:**
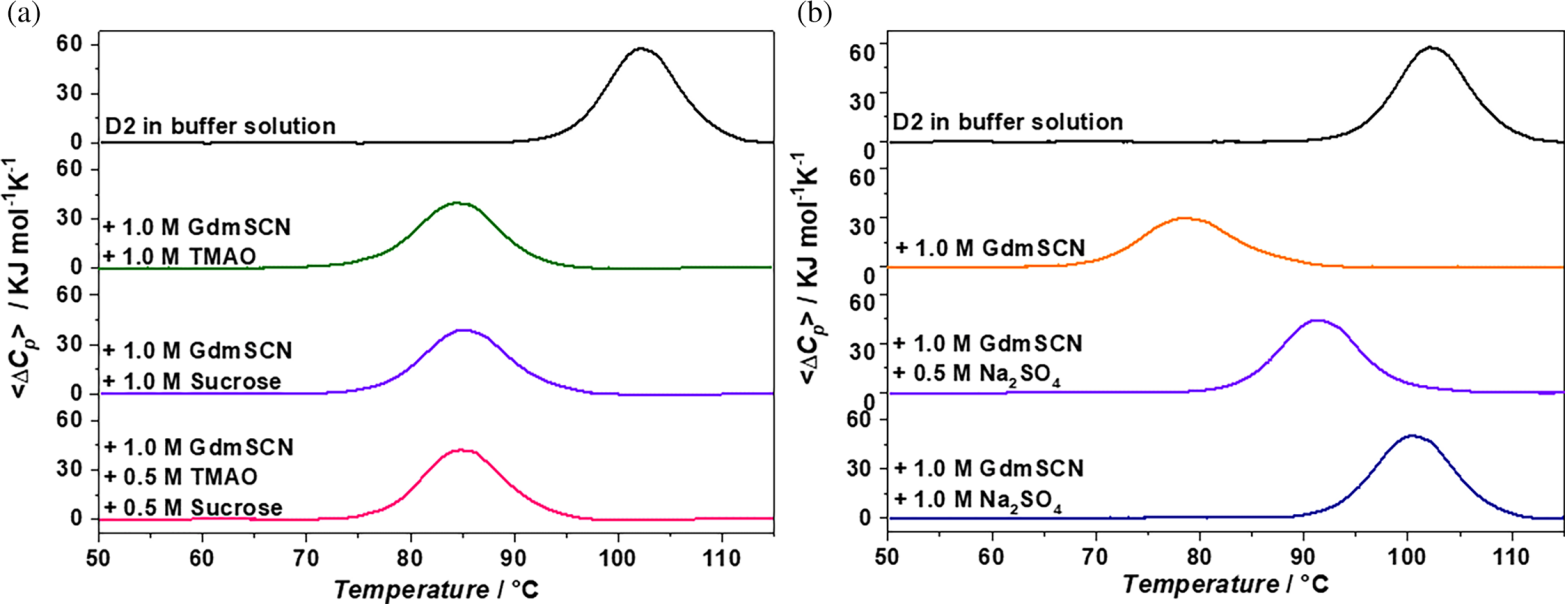
DSC traces of D2 in aqueous 20 mM phosphate buffer, pH 7.4, and in the presence of 1 M GdmSCN and 1 M of different cosolutes (a); DSC traces of D2 in aqueous 20 mM phosphate buffer, pH 7.4, and in the presence of 1 M GdmSCN and different concentrations of sodium sulfate (b).

Counteraction means that the native state destabilization caused by a denaturant is reversed by the addition of a stabilizing agent or viceversa. Some of us have shown that counteraction is exerted by all the stabilizing agents against the native state destabilization caused by all the denaturing agents (Cozzolino et al., 2018; Cozzolino et al., 2021; Vigorita et al., 2018); so it is possible to use the expression general counteraction. To study counteraction in the present case, DSC measurements have been performed on the D2 domain dissolved in an aqueous solution containing both 1 M GdmSCN and 1 M of some selected stabilizing agents (see panels A and B of Figure 9). Specifically, 1 M TMAO, 1 M sucrose, 0.5 M TMAO +0.5 M sucrose, and 0.5 M and 1 M Na_2_SO_4_ have been tested. In all such conditions, the temperature‐induced denaturation is reversible, according to the re‐heating criterion (see Figures S3 and S4), and well‐described by the reversible two‐state transition model (see the values in the last column of Table 1). Data indicate that the addition of 1 M TMAO, or 1 M sucrose, or 0.5 M TMAO +0.5 M sucrose to an aqueous solution containing 1 M GdmSCN causes a significant *T*_*d*_ increase, from 78.4°C (i.e., the *T*_*d*_ value in 1 M GdmSCN) to about 85°C. It is interesting to note that the effect of TMAO and sucrose proves to be additive on the denaturation temperature, suggesting that the counteraction mechanism cannot be due to direct interactions of the stabilizing agents with the protein surface. Moreover, *T*_*d*_ = 99.6°C in a solution containing both 1 M GdmSCN and 1 M Na_2_SO_4_, confirming that sodium sulfate is a stabilizing agent stronger than TMAO and sucrose (Cozzolino et al., 2018; Vigorita et al., 2018); this should not be a surprise considering that sulfate is one of the strongest salting‐out anions (Hofmeister, 1888; Zhang & Cremer, 2010). Since the values of the denaturation enthalpy change do not increase, but remain constant, in contrast to the rise of the denaturation temperature values (look at the numbers in part D of Table 1), one must conclude that general counteraction is basically of entropic origin. This is an important result because it provides additional information with respect to the Gibbs free energy change obtained by studying the action of stabilizing or destabilizing agents and their mixtures in isothermal conditions. A rationalization of this result cannot be grounded in the elegant approaches developed by Schellman (Schellman, 2003), Bolen (Auton et al., 2011) and Record (Record et al., 2013) that solely refer to the Gibbs free energy changes caused by the preferential exclusion or accumulation of cosolutes in the protein solvation shell.

A reliable rationalization of the latter has been provided by some of us (Cozzolino et al., 2018; Cozzolino et al., 2021; Vigorita et al., 2018). In all liquids, it is necessary to create a cavity to host a solute molecule, paying a Gibbs free energy cost (Graziano, 2006). The latter comes from the solvent‐excluded volume effect: the reduction in the number of spatial configurations accessible to liquid molecules as a consequence of cavity creation (Bologna & Graziano, 2023; Graziano, 2006). Moreover, the magnitude of the solvent‐excluded volume effect increases with the liquid number density (i.e., this is why it is larger in water in comparison to common organic liquids), which is proportional to the density of the considered aqueous solutions (Graziano, 2006). Importantly, keeping fixed the cavity van der Waals volume, the Gibbs free energy cost of cavity creation increases with the solvent‐accessible surface area, SASA, of the cavity itself (Graziano, 2015) (i.e., on passing from a spherical to a prolate spherocylindrical shape, for instance). This holds because a cavity can exist solely if the center of liquid molecules cannot stay within the volume enclosed by the cavity SASA (Bologna & Graziano, 2023; Graziano, 2006; Graziano, 2015). In the case of proteins, it is necessary to recognize that (Cozzolino et al., 2018; Pica & Graziano, 2016; Vigorita et al., 2018): (a) the cavity shape has to be different on passing from the native state to the denatured one; (b) the two protein states have practically the same molecular volume (Chalikian, 2003; Chen & Makhatadze, 2017; Royer, 2002), but a markedly different SASA; (c) the Gibbs free energy cost of cavity creation is markedly larger for the denatured state than for the native state, providing a significant thermodynamic stabilization of the latter; (d) this thermodynamic stabilization rises on increasing the density of the aqueous solutions and this is what happens for aqueous solutions containing both a destabilizing agent and a stabilizing one (Cozzolino et al., 2018; Cozzolino et al., 2021; Vigorita et al., 2018); for instance, at 25°C, the density of water is 997 g l^−1^, that of 1 M GdmSCN is 1023 g l^−1^, that of 1 M sucrose is 1126 g l^−1^, and that of 1 M GdmSCN +1 M sucrose is 1154 g l^−1^. Clearly, the solvent‐excluded volume effect is not the entire story. The direct binding to protein surfaces of denaturants and the associated increase in the number of configurational microstates (i.e., the contributions favoring the denatured state), have to be accounted for (Cozzolino et al., 2018; Cozzolino et al., 2021; Paladino, Balasco, Graziano, & Vitagliano, 2022; Paladino, Balasco, Vitagliano, & Graziano, 2022; Vigorita et al., 2018). However, it appears that the increase in the magnitude of the solvent‐excluded volume effect is dominant, leading to a T_d_ increase (Cozzolino et al., 2018; Cozzolino et al., 2021; Vigorita et al., 2018). Based on MD simulations (Bennion & Daggett, 2004; Kokubo et al., 2011), it was shown that the excess of urea molecules on the surface of peptides and proteins is not disturbed by the addition of TMAO; similarly, it is possible to guess that thiocyanate or guanidinium ions do not leave the protein surface to go to the bulk of the aqueous solution upon the addition of stabilizing agents. Even though somehow expected, present results confirm that counteraction is operative also for a hyper‐thermophilic globular protein.

## 3 CONCLUSIONS

Although structurally and functionally integrated into the global structure of the parent protein, the D2 domain of TmArgBP retains notable properties such as extraordinary stability against temperature and pressure (Jaworek et al., 2020; Smaldone et al., 2018). Since the temperature‐induced denaturation of this domain is a two‐state reversible process, it represents a suitable model system to perform investigations focused on the conformational stability of a hyper‐thermophilic protein.

The denaturing action of the thiocyanate ion on the D2 domain has been dissected at various levels, via experimental and computational approaches. The destabilization induced by the thiocyanate ion, which is further enhanced by adding the guanidinium ion (GdmSCN), is mainly associated with direct interactions of these ions with protein groups, and is characterized by both energetic and entropic contributions. DSC experiments performed in the presence of several cosolutes show that the destabilization is promptly rescued by the addition of stabilizing agents. Moreover, MD results confirm the promiscuous nature of both guanidinium and thiocyanate ions in mediating interactions with all kinds of protein moieties, thus offering a mechanistic description of the denaturing process.

In conclusion, the present findings corroborate and expand previous observations (Cozzolino et al., 2018; Cozzolino et al., 2020; Cozzolino et al., 2021; Smaldone et al., 2018), indicating that key mechanisms, such as those underlying the counteraction effects exerted by stabilizing agents (Karshikoff et al., 2015; Pica & Graziano, 2016; Vigorita et al., 2018), are valid for all proteins.

## 4 MATERIALS AND METHODS

### 4.1 Protein, materials and sample preparation

The D2 domain (residues 115–206) of TmArgBP, carrying a polyhistidine‐tag at its C‐terminus, was recombinantly expressed and purified as previously described (Smaldone et al., 2018). All chemicals were of analytical grade and used without further purifications; urea, TMAO (trimethylamine N‐oxide), sucrose, and sodium sulfate were from Sigma; GdmSCN was from Fluka. A 20 mM sodium phosphate buffer solution at pH 7.4 was used for sample preparation. Urea stock solutions were freshly prepared by weight in a 3 ml final volume calibrated flask. GdmSCN was purchased as a ready‐to‐use 6 M buffered aqueous solution. Sucrose, TMAO, and sodium sulfate stock solutions were prepared by weight in a 10 ml final volume calibrated flask. Before calorimetric measurements, D2 samples were dialyzed in PBS solution at pH 7.4, and their concentration after dialysis was obtained by measuring the absorbance at 280 nm and using a theoretical molar extinction coefficient (Pace et al., 1995).

### 4.2 Differential scanning calorimetry

DSC measurements were performed using a Nano‐DSC (TA Instruments, Delaware) operating with two capillary cells of 300 μl sensitive volume and kept at the total pressure of 3 atm during the scan. DSC thermograms were recorded by monitoring the difference in the heat capacity of the protein solution upon increasing temperature from 25°C to at most 115°C at a scan rate of 1 °C/min, followed by cooling and subsequent re‐heating of the sample at the same scan rate to the same final temperature. Reproducibility and reversibility of temperature‐induced denaturation were confirmed by comparing the first and second heating scans after cooling for each sample. Protein samples were prepared at the concentration of 0.5 mg/ml in the 20 mM Na phosphate buffer at pH 7.4. The buffer‐buffer scan was subtracted from the protein solution scan for each sample, and the excess molar heat capacity function <Δ*C*_*p*_> was obtained after the baseline subtraction. These data were analyzed using the Nano‐Analyze software (TA Instruments) and plotted using the Origin software package to obtain the thermodynamic parameters associated with denaturation. The denaturation temperature, *T*_*d*_, corresponds to the maximum of the DSC profile; the calorimetric enthalpy change, Δ*H*_*d*_(*T*_*d*_), is the total integrated area below the DSC peak after baseline subtraction, indicative of the total heat energy uptake by the sample; the van't Hoff enthalpy change, Δ*H*_*d*_(*T*_*d*_)^vH^, is an independent measure of the transition enthalpy change, according to the assumed model for the process. Assuming a simple two‐state model, the van't Hoff enthalpy change is calculated employing a well‐established formula (Privalov, 1979). The ratio between the calorimetric enthalpy and the effective van't Hoff enthalpy, Δ*H*_*d*_(*T*_*d*_)/Δ*H*_*d*_(*T*_*d*_)^vH^, must be close to one to state that protein unfolds as a single cooperative domain (Privalov, 1979; Zhou et al., 1999).

### 4.3 Isothermal titration calorimetry

ITC measurements were performed using a Nano‐ITC III (TA instruments, Delaware) operating at the temperature of 25°C. Briefly, a solution of the protein, at the concentration of 42 μM, was placed in the calorimeter vessel (final volume of 961 μl); while a KSCN solution (300 mM) was loaded in a 250 μl volume syringe. The titration was performed by injecting 25 aliquots of 10 μl of KSCN in the calorimeter vessel with 400 s intervals between the individual injections. In order to evaluate the heat of dilution of KSCN, the same experiment was also performed by titrating the salt solution in the calorimeter vessel containing the phosphate buffer. The obtained heat peaks were integrated using Nano Analyze software supplied with the instrument. The binding isotherm was obtained by plotting the cumulative heat versus the total concentration of KSCN. The obtained data were fit with a model of equal and independent binding sites, which allowed the determination of the binding constant and the binding enthalpy change using the following equation (Oliva et al., 2015):

$$\sum_{k=1}^{k}∆h_{k}=\left[\frac{1}{\left[P_{0}\right]}\left(-\frac{\sqrt{b^{2}-4\mathrm{ac}}-b}{2a}\right)\right]\sum_{k=1}^{n}∆h_{k}$$

where $a=nK_{b};b=\left(1+n\left[P_{0}\right]K_{b}+\left[L_{0}\right]K_{b}\right);c=\left[L_{0}\right]\left[P_{0}\right]K_{b}.$ In this equation, *K*_b_ is the binding constant, *n* is the stoichiometry (number of ligand SCN^−^ ions bound per molecule of protein), [*P*_0_] and [*L*_0_] are the protein and KSCN total concentration, respectively. Instead, $\sum_{k=1}^{k}∆h_{k}$ and $\sum_{k=1}^{n}∆h_{k}$ are the cumulative heats of the first k injections and the total heat of injections, respectively. The value of $\sum_{k=1}^{n}∆h_{k}$ represents the binding enthalpy change.

### 4.4 Molecular dynamics simulations

MD studies were performed using the Gromacs software package (version 2020.3) with the charmm27 force field (Bjelkmar et al., 2010; van der Spoel et al., 2005). The crystal structure of the D2 domain from TmArgBP has been used as a starting model for MD studies (PDB entry: 6 gpm) (Smaldone et al., 2018). Three different simulation systems were generated: TmArgBP in pure water (44,183 water molecules), TmArgBP in 2 M KSCN aqueous solution (39,017 water and 1500 KSCN molecules) and TmArgBP in 2 M GdmSCN (41,180 water molecules and 1500 GdmSCN). Force field parameters for the thiocyanate and guanidinium ions were obtained from (Camilloni et al., 2008; Tesei et al., 2018). The 92 residue protein domain was centered in a triclinic box at a 1.0 nm distance from each box edge and solvated in the SPCE water model (Berendsen et al., 1987). Counterions (Cl^−^, K^+^) were randomly added to neutralize the system, and periodic boundary conditions were applied in the three dimensions. A cutoff radius of 0.9 nm for nonbonded van der Waals interactions was used in all simulations. Bond lengths involving hydrogens were restrained by the LINCS algorithm (Hess et al., 1997). Electrostatic interactions were treated using the particle mesh Ewald method (Darden et al., 1993). An integration time step of 2 fs was used. The system was energy minimized using the steepest descent approach, followed by an equilibration phase in the NVT ensemble (temperature coupling was kept by the velocity rescale thermostat scheme), and an additional 100 ps equilibration step in the NPT ensemble, by coupling the pressure with a Parrinello‐Rahman barostat (Parrinello & Rahman, 1981). After equilibration, MD production runs of 200 ns *per* system were carried out in the NPT ensemble (T = 300 K, *p* = 1.0 bar). To enhance sampling, five independent replicas with different initial velocities were run for each system (1 μs × system × temperature). Additionally, to increase protein structural fluctuations and observe starting events of thermal denaturation of D2 in 2 M KSCN, elevated temperatures were also employed (i.e., *T* = 350 and 400 K). It is important to underscore that, in the considered timescales, high‐temperature simulations are meant to reproduce only local structural adjustments that could be observed in the initial stages of the unfolding upon addition of denaturants. Analyses of the MD trajectories were carried out using GROMACS routines and the VMD program (Humphrey et al., 1996).

## AUTHOR CONTRIBUTIONS

**Guido Izzi:** Investigation; formal analysis. **Antonella Paladino:** Investigation; formal analysis; visualization; writing – original draft; writing – review and editing; funding acquisition; data curation. **Rosario Oliva:** Methodology; supervision. **Giovanni Barra:** Investigation. **Alessia Ruggiero:** Methodology; supervision. **Pompea del Vecchio:** Supervision; data curation; methodology; writing – original draft. **Luigi Vitagliano:** Conceptualization; writing – original draft; writing – review and editing; data curation. **Giuseppe Graziano:** Conceptualization; funding acquisition; writing – original draft; writing – review and editing; data curation; project administration.

## Supporting information


**DATA S1.** Supporting information includes Figure S1 showing the crystallization trials carried out on the D2 domain of ArgBP grown in the presence of 2 M SCN^−^; Figure S2 displaying the radius of gyration and root mean square fluctuations *per* residues of D2 along the 5 MD simulations at 300 K in water and in 2 M KSCN; Figures S3 and S4 showing the reversibility of the temperature‐induced denaturation of D2 in the presence of 1 M GdmSCN plus 1 M sucrose or 1 M sodium sulfate.
