# Supplementary material for: Destabilization of the D2 domain of Thermotoga maritima arginine binding protein induced by guanidinium thiocyanate and its counteraction by stabilizing agents
Source: Protein Sci. 2024 Aug 16;33(9):e5146. doi: 10.1002/pro.5146 (PMC11328109; doi:10.1002/pro.5146)
Supplement: Supplementary file 1 — DATA S1. Supporting information includes Figure S1 showing the crystallization trials carried out on the D2 domain of ArgBP grown in the presence of 2 M SCN−; Figure S2 displaying the radius of gyration and root mean square fluctuations per residues of D2 along the 5 MD simulations at 300 K in water and in 2 M KSCN; Figures S3 and S4 showing the reversibility of the temperature‐induced denaturation of D2 in the presence of 1 M GdmSCN plus 1 M sucrose or 1 M sodium sulfate. [file PRO-33-e5146-s001.docx]

**Supporting Information**

**Destabilization of the D2 domain of *Thermotoga maritima* Arginine Binding Protein induced by guanidinium thiocyanate and its counteraction by stabilizing agents**

Guido Izzi,a# Antonella Paladino,b# Rosario Oliva,a Giovanni Barra,b Alessia Ruggiero,b Pompea Del Vecchio,a Luigi Vitagliano,b Giuseppe Grazianoc,*

**Figure S1.** A typical crystal of the D2 domain of ArgBP grown in the presence of 2 M SCN-. Crystallization trials were performed at a protein concentration of 14 mg ml−1 in a solution medium containing 0.1 M Bis-Tris (pH 6.5) and 28% (w/v) polyethylene glycol 2000. The length of the crystal is ~ 0.3 mm.


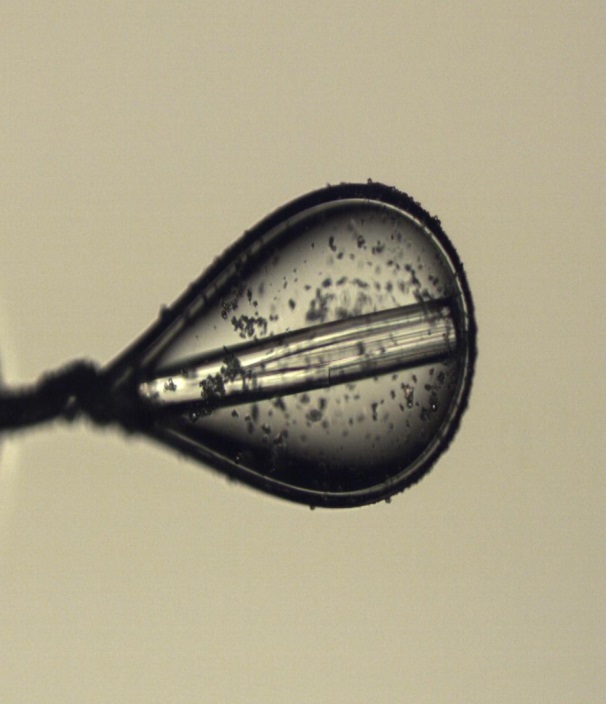


**Figure S2.** Values of the D2 radius of gyration (Rg) along the 5 MD trajectories (r1-5) at 300 K in water (panel A) and in 2 M KSCN (panel B); red line at 10.8Å corresponds to the Rg value in the D2 X-ray structure. Evolution of the root mean square fluctuation *per* residue along the simulation time in both solution buffers (i.e., water and 2 M SCN) at 300K (panel C).


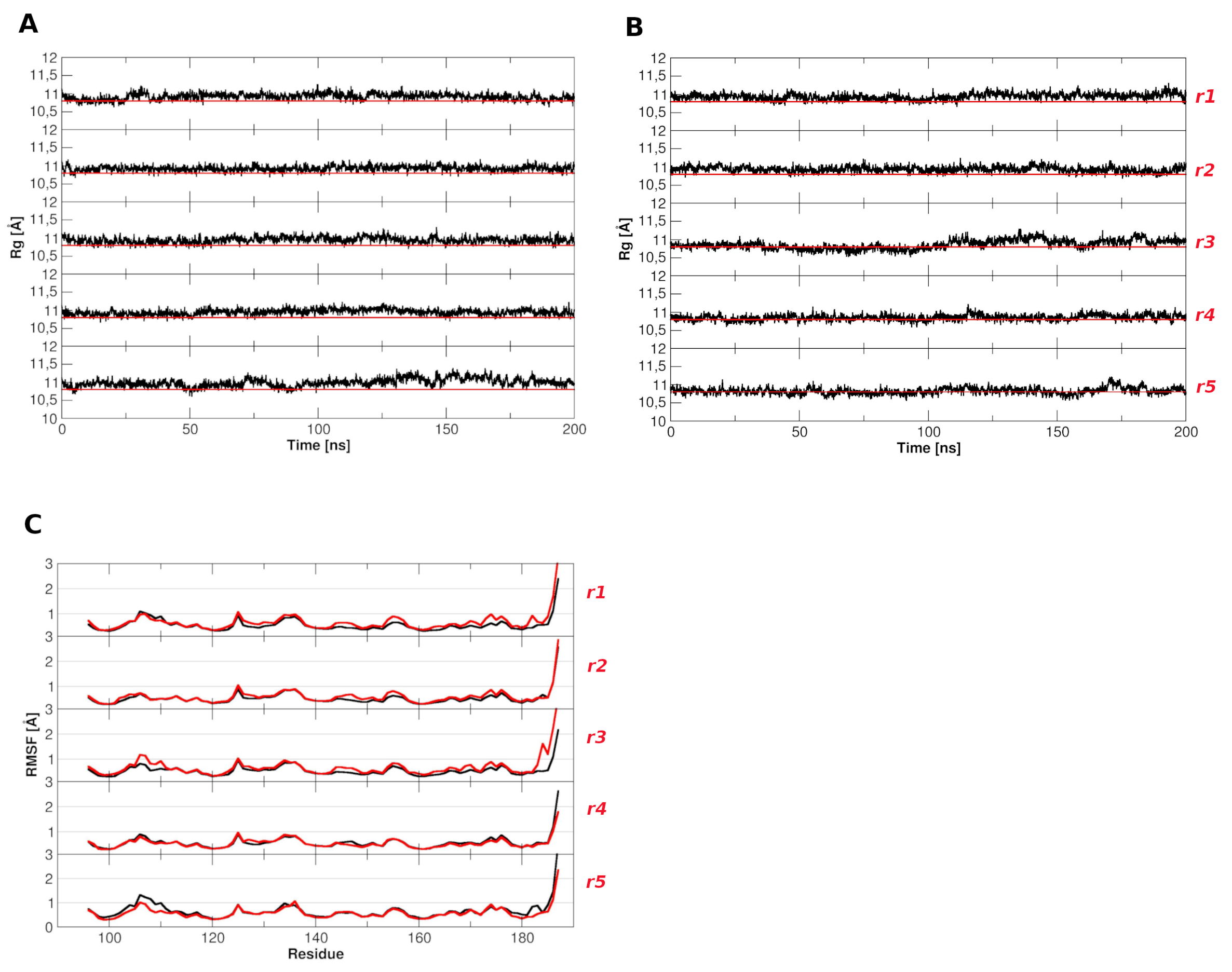


**Figure S3**. DSC profiles of the first heating (black solid line), second heating (red dashed line) and third heating (blue dotted line) for D2 in buffer solution containing 1M sucrose.

**Figure S4.** DSC profiles of the first heating (black solid line) and second heating (red dashed line) of D2 in buffer solution containing 1 M GdmSCN + 1 M sodium sulphate.
